# Persistence of Body Composition Changes Observed During the Winter Holiday Period: A Three-Time-Point, One-Year Longitudinal Study

**DOI:** 10.3390/medicina62030511

**Published:** 2026-03-10

**Authors:** Ion-Vladut Udroiu, Alin Albai, Sandra Lazar, Adina Braha, Laura Gaita, Bogdan Timar, Alexandra Sima

**Affiliations:** 1 Doctoral School of Medicine, “Victor Babes” University of Medicine and Pharmacy, 300041 Timisoara, Romania; ion-vladut.udroiu@umft.ro (I.-V.U.); sandra.lazar@umft.ro (S.L.); 2 Department of Diabetes, “Pius Brinzeu” Emergency Hospital, 300723 Timisoara, Romania; braha.adina@umft.ro (A.B.); gaita.laura@umft.ro (L.G.); bogdan.timar@umft.ro (B.T.); sima.alexandra@umft.ro (A.S.); 3 Centre for Molecular Research in Nephrology and Vascular Disease, “Victor Babes” University of Medicine and Pharmacy, 300041 Timisoara, Romania; 4 Second Department of Internal Medicine, “Victor Babes” University of Medicine and Pharmacy, 300041 Timisoara, Romania; 5 First Department of Internal Medicine, “Victor Babes” University of Medicine and Pharmacy, 300041 Timisoara, Romania; 6 Department of Hematology, Emergency Municipal Hospital, 300254 Timisoara, Romania

**Keywords:** body composition, winter holidays, bioimpedance analysis, weight gain, visceral fat

## Abstract

*Background and Objectives*: Weight gain during winter holidays has been reported in several studies, but most of them focus on short-term changes and rely primarily on body weight or BMI. This study investigates if body composition alterations associated with winter holiday period persist over a one-year follow-up in a Romanian adult population. *Materials and Methods*: This prospective longitudinal observational study included three assessment points: before the winter holidays (T1), immediately after the holidays (T2), and one year later (T3). Bioelectrical impedance analysis was used to obtain body composition parameters. A total of 120 participants completed all three assessments and were included in the longitudinal analysis. *Results*: Body weight and visceral fat area increased modestly yet significantly between T1 and T2. At the one-year follow-up, values remained similar to those observed immediately after the holiday period, suggesting persistence at group level (body weight: 68.25 → 69.40 → 69.45 kg and visceral fat area: 98.49 → 100.54 → 101.42 cm^2^). The net change observed during the holiday period was similar in magnitude to the overall annual difference. Changes in body weight were significantly associated with changes in visceral fat both during the holiday period and across the entire follow-up. *Conclusions*: Modest increases in body weight and visceral fat observed during the winter holidays were still present at one year. These findings suggest that short seasonal periods may contribute to overall annual changes in body weight and fat distribution.

## 1. Introduction

Over recent decades, obesity prevalence increased despite continuous research, increased awareness, and modern treatments. Projections indicate that by 2030, around half of all adults will be overweight and one in five will be obese [1,2]. This trend is concerning given the strong relationship between obesity and cardiometabolic risk [2,3,4,5]. The distribution of adipose tissue determines the severity of this risk [5,6]. Compared with subcutaneous adipose tissue (SAT), visceral adipose tissue (VAT) plays a much more harmful role. It is linked to insulin resistance, dyslipidemia, inflammation, atherosclerosis, prothrombotic profile and other cardiometabolic disturbances [6,7,8].

The development of obesity is complex and influenced by multiple factors. Although genetics determines an individual’s predisposition, excessive weight is mainly the result of an mismatch between caloric intake and energy expenditure [9]. The main contributing factors are high-calorie diets and reduced physical activity, further influenced by factors such as socioeconomic status, environment, stress, certain medications, and medical conditions [10].

Beyond the causes of obesity, patterns of weight change over time may be equally relevant. Epidemiological data from large longitudinal cohorts show that adult weight gain is usually modest, averaging about 0.5–1 kg per year. In the CARDIA study, annual weight gain ranged from 0.55 to 0.96 kg across groups, leading to mean increases of 14–21 kg over 25 years [11,12,13]. Similar patterns have been reported in other prospective cohorts [14]. These observations raise an important question. Does long-term weight gain reflect a continuous process or is the net result of repeated cycles of weight gain and incomplete weight loss?

Seasonal fluctuations in body weight appear to play an important role. Periods characterized by increased caloric intake, changes in dietary composition, reduced physical activity, and disruption of daily routines are consistently associated with short-term positive energy balance [15,16]. A period when all these factors converge is the end of the year. In this period multiple cultural and social holidays are celebrated across the world. Despite consistent evidence of seasonal weight gain, most studies have focused primarily on body weight or body mass index (BMI). These measures do not capture alterations in body fat distribution, and they can remain relatively stable even when fat mass and fat distribution change [17,18,19]. For this reason, assessing body composition and adiposity may add relevant information beyond weight alone.

In this context, evaluating body fat distribution is important consdering that is a critical marker for cardiometabolic risk. Both SAT and VAT are associated with metabolic risk [20,21]. However, VAT shows a stronger and more independent relationship with adverse metabolic outcomes, even after adjustment for anthropometric indices [20]. VAT may respond to changes in energy balance and does not always parallel changes in overall body weight [21,22,23].

Although seasonal weight gain has been described in several countries, most studies measured participants only before and shortly after the holiday period. This design does not show if these short-term changes persist over time or what magnitude this short period has compared with the net annual change. Longitudinal data on visceral adiposity across a full year are still limited. Results also differ between countries, probably due to differences in cultural habits, diet, and holiday duration. To our knowledge, no study in a Romanian adult population has examined whether holiday-related changes in body weight and body composition persist over the following year. To address these limitations, the present study evaluated body weight and body composition derived from bioelectrical impedance analysis (BIA) at three time points across one year, allowing assessment of both short-term holiday-related changes and their persistence over time, as well as their magnitude in relation to the net annual change.

Considering these aspects, the main objective of the present study was to investigate if alterations in body weight and body composition, especially visceral fat area (VFA) derived from BIA, observed during the winter holiday period persist over a one-year follow-up. In addition, we aimed to evaluate the magnitude of these holiday-related changes in relation to the net annual variation in body weight and body composition.

We hypothesized that the increases in body weight and VFA observed during the winter holiday period would persist at one year and would contribute to the net annual change.

## 2. Materials and Methods

A longitudinal, prospective observational study was conducted with three assessment points: before the winter holidays (T1), immediately after the winter holidays (T2), and at one-year follow-up (T3). Short-term changes between T1 and T2 have been reported previously [24]. In the present analysis, these changes were reassessed in the current cohort and participants were followed from T2 to T3 to describe the evolution of body composition after the winter holiday period. The baseline assessment (T1) was performed in early December, before the winter holidays. T2 was conducted in January, immediately after the holiday period. T3 was performed one year later, in the second half of November, prior to the next winter holiday season.

All assessments were carried out at the Center for Diabetes, Nutrition and Metabolic Diseases, part of the Emergency County Hospital “Pius Brînzeu”, Timișoara, Romania. Participants were recruited voluntarily through social media announcements. Individuals who expressed interest simply contacted the study team, representing a convenience sample of adults from the local community. Eligibility criteria included age ≥ 18 years. Exclusion criteria included pregnancy and the presence of implanted electronic devices. No additional restrictions were applied regarding metabolic status or comorbidities.

A total of 168 participants completed T1 and T2 assessments. Because T3 was conducted one year later, 120 (71.4%) of these participants returned for the follow-up, corresponding to a loss of 48 participants (28.6%). To evaluate potential attrition bias, baseline demographic and body composition characteristics were compared between completers and dropouts using independent-sample tests for continuous variables and chi-square test for sex comparison (Supplementary Table S1). The 120 participants who attended all three assessments were included in the longitudinal analyses presented in this study. Written informed consent was obtained from all individuals. The study received approval from the Ethics Committee of the Emergency County Hospital “Pius Brînzeu”, Timișoara, Romania (508/25 November 2024) and was conducted aligned with the Declaration of Helsinki (latest revision). Participant confidentiality was ensured throughout the study in compliance with General Data Protection Regulation (GDPR) requirements.

Anthropometric and body composition measurements were performed at all three assessment points. A BIA analyzer (InBody 770, InBody Co., Ltd., Seoul, South Korea) was used to obtain body composition parameters. One important estimated parameter provided by this device is VFA. It is derived from proprietary multifrequency BIA prediction algorithms and does not represent direct imaging of visceral adipose tissue (e.g., CT or MRI). Previous validation studies have reported moderate correlations between BIA derived and CT measured VFA (r ≈ 0.65–0.76), with intraclass correlation coefficients (ICC) around 0.67, indicating moderate agreement [25,26]. The InBody 770 has demonstrated very high test–retest reliability under standardized conditions (ICC ≥ 0.99) and low day-to-day biological variability [27]. Multifrequency BIA has been shown to detect pre–post changes without significant differences compared to reference methods [25,28]. In this study, all assessments were performed using the same device and standardized conditions (fasting state, controlled hydration, same time of day), aiming to minimize within-subject variability. Pegaso stadiometer (GIMA S.p.A., Gessate, Italy) was used to obtain the height of the participants. A flexible measuring tape was used to measure waist circumference (WC) and hip circumference (HC) according to World Health Organization (WHO) recommendations [29]. Waist-to-hip ratio (WHR) and waist-to-height ratio (WHtR) were subsequently calculated. Additional variables related to holiday behaviors (including self-reported dietary patterns and residential environment during the holiday period) were collected and analyzed in the previously published T1–T2 study [24]. The present analysis focuses specifically on the longitudinal persistence of body composition changes and does not re-examine these behavioral variables.

Data analysis was conducted with MedCalc^®^ Statistical Software (version 23.4.3; MedCalc Software Ltd., Ostend, Belgium). A *p*-value threshold of 0.05 was used for statistical significance, corresponding to a 95% confidence level. A sensitivity analysis was performed to quantify the minimum detectable effect (MDE) for the paired comparisons. With n = 120, α = 0.05 (two-tailed) and 80% power, the study can detect a standardized paired effect of approximately 0.26 SD of the paired differences. Based on the observed SD of Δ (T1–T2), the corresponding MDE was approximately 0.39 kg for body weight, 1.69 cm^2^ for VFA, and 0.31 kg for body fat mass (BFM). The Shapiro–Wilk test was used to test the normality of continuous variables. Continuous data are reported as mean ± standard deviation (SD) for variables with a normal distribution and as median (interquartile range, IQR) for variables with a non-normal distribution. Changes in body composition and anthropometric parameters over time were analyzed using paired statistical tests. Depending on data distribution, for two time point comparisons (T1–T2 and T2–T3) parametric or non-parametric tests were used. To evaluate longitudinal changes across all three assessment points (T1–T2–T3), repeated-measures analyses were performed (repeated-measures ANOVA with Greenhouse–Geisser correction or the Friedman test, depending on the distribution of data). Relationships between changes in body weight (ΔWeight) and changes in visceral fat area (ΔVFA) were evaluated using correlation analyses. Depending on variable distribution, Pearson or Spearman methods were used. Simple linear regression was used to further examine these associations. To examine weight trajectories after the winter holiday period, participants were categorized based on changes in body weight between T2 and T3: weight loss greater than 1 kg, weight stability within ±1 kg, or weight gain more than 1 kg. Baseline characteristics were compared across these groups. Continuous variables were analyzed using the Kruskal–Wallis test and categorical variables were analyzed using chi-square methods.

## 3. Results

Table 1 presents the demographic and body composition characteristics of the study participants. The three assessment points were completed by 120 participants with a median age of 30 years (IQR 26–43.5) and a mean height of 167.95 ± 9.01 cm. Baseline characteristics did not differ significantly between completers and dropouts (all *p* > 0.05; Supplementary Table S1).

Table 2 shows demographic and body composition characteristics at T1 stratified by sex. The study included 86 females (71.7%) and 34 males (28.3%). As expected, males were significantly taller and heavier than females. Consistent with these differences, male participants exhibited higher lean tissue parameters [including fat free mass (FFM), skeletal muscle mass (SMM)] and higher parameters in body water compartments [total body water (TBW), intracellular water (ICW) and extracellular water (ECW) (all *p* < 0.001)]. Percentage of body fat (PBF), BFM and VFA were higher in female participants. Several anthropometric indices, including WC, HC, WHR, and WHtR, also differed significantly between sexes.

Acute changes between T1 and T2 are presented in Table 3. Between T1 and T2, statistically significant increases were observed in body weight [68.25 → 69.40 kg (*p* = 0.015)] and BMI [24.3 → 24.4 kg/m^2^ (*p* = 0.018)]. Measures of adiposity also increased, with both BFM [21.09 → 21.43 kg (*p* = 0.002)] and PBF [29.83% → 30.16% (*p* = 0.009)] changing. Indicators of visceral adiposity also showed increases, as reflected by VFA [98.49 → 100.54 cm^2^ (*p* < 0.001)] and visceral fat level (VFL) [8.5 → 9 (*p* = 0.009)]. WC increased [83.36 → 84.09 cm (*p* < 0.001)] and HC changed (*p* = 0.006), resulting in statistically significant increases in both WHR [0.823 → 0.827 (*p* = 0.02)] and WHtR [0.496 → 0.5 (*p* < 0.001)]. No statistically significant changes were observed in lean tissue parameters or body water compartments. Given the unequal sex distribution and baseline differences, additional analyses were performed to compare the magnitude of holiday-related changes (T1–T2) between females and males. No statistically significant sex differences were observed for changes in VFA or other body composition parameters (all *p* > 0.05). Detailed results are provided in Supplementary Table S2.

Table 4 shows the changes observed between T2 and T3. No statistically significant changes were observed in body weight or BMI. Visceral adiposity indices (VFA and VFL), BFM, PBF had no statistical significance difference between the follow up. SMM increased between T2 and T3 [from 25.1 to 25.2 kg (*p* = 0.044)]. No statistically significant changes were observed in FFM, body water compartments, or anthropometric indices (WC, HC, WHR, WHtR) during the follow-up interval. Additional sex-specific comparisons were performed for the T2-T3 interval. No statistically significant differences between females and males were observed in the magnitude of changes in body weight, VFA, or other body composition parameters (all *p* > 0.05; Supplementary Table S3).

Exploratory Spearman correlation analyses showed no significant associations between age and changes in body weight or VFA, either during the holiday period (ΔWeight T1–T2: ρ = −0.05, *p* = 0.589; ΔVFA T1–T2: ρ = −0.076, *p* = 0.412) or across the one-year follow-up (ΔWeight T2–T3: ρ = −0.028, *p* = 0.761; ΔVFA T2–T3: ρ = −0.091, *p* = 0.321).

**Figure 1:**
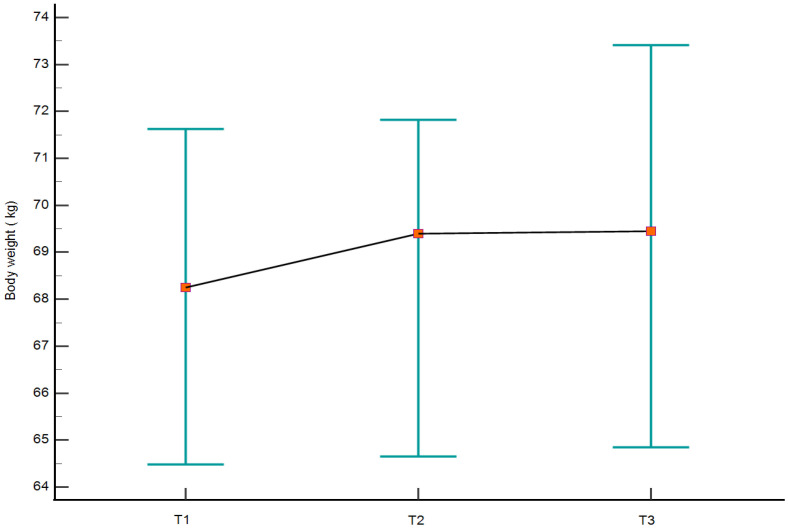
Longitudinal body weight changes across the three assessment points (T1, pre-holiday; T2, post-holiday; T3, one-year follow-up). Body weight is presented as median values (square markers) with 95% confidence intervals for the median (vertical bars). The solid line connects the central tendency values across time points to illustrate the overall trajectory.

**Figure 2:**
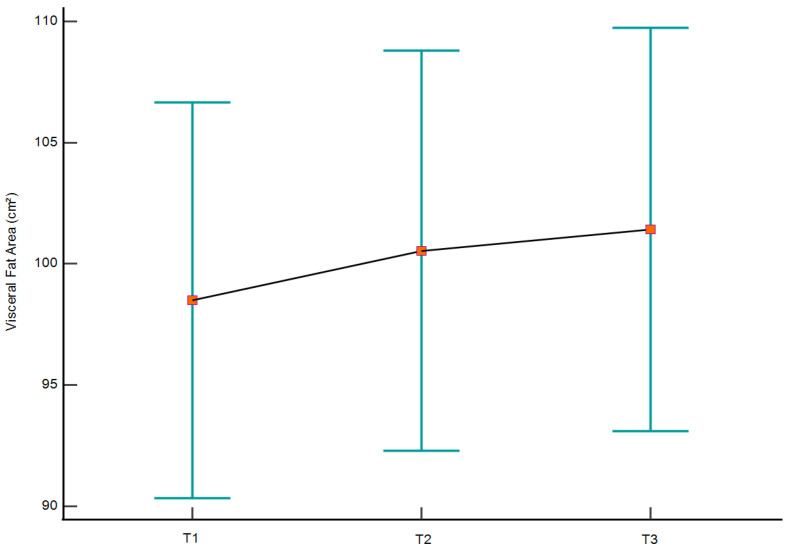
Longitudinal changes in visceral fat area (VFA) across the three assessment points (T1, pre-holiday; T2, post-holiday; T3, one-year follow-up). VFA is presented as mean values (square markers) with 95% confidence intervals for the mean (vertical bars). The solid line connects the central tendency values across time points to illustrate the overall trajectory.

Longitudinal analyses for the primary outcomes across the three assessment points are summarized in Table 5 and illustrated in Figure 1 and Figure 2. Body weight showed an increase from T1 to T2, followed by stabilization at T3, with no statistically significant overall effect of time (*p* = 0.122). In contrast, repeated-measures ANOVA revealed significant longitudinal effects for both BFM (*p* = 0.020) and VFA (*p* = 0.025). These parameters had increases from baseline that persisted at follow-up, with no statistical significant differences between T2 and T3. The longitudinal trajectories are visually depicted in Figure 1 and Figure 2. Longitudinal analyses of the remaining body composition and anthropometric parameters are presented in Supplementary Table S4.

Participants were grouped according to weight changes between T2 and T3 to describe post-holiday follow-up trajectories: weight loss (<−1 kg), weight maintenance (±1 kg), and further weight gain (>+1 kg). Between T2 and T3, 34.2% of participants maintained their post-holiday body weight within ±1 kg, 36.7% experienced further weight gain, and 29.2% showed partial weight loss. Table 6 summarizes baseline characteristics across these post-holiday categories. Across these groups, baseline age, sex distribution, body weight, BFM, and VFA did not differ statistically significant.

**Figure 3:**
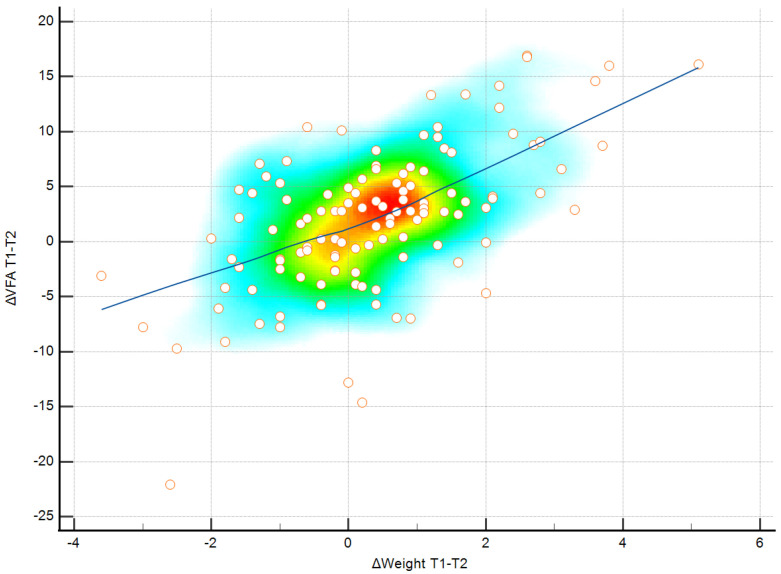
Pearson correlation between changes in visceral fat area (ΔVFA) and changes in body weight (ΔWeight) during the winter holiday period (T1–T2). Color shading reflects observation density, individual values are shown as points, and the blue line represents the linear association.

The relationship between ΔWeight and ΔVFA during the winter holiday period was examined using Pearson correlation. As shown in Figure 3, ΔWeight was positively correlated with ΔVFA(r = 0.608, *p* < 0.001).

**Figure 4:**
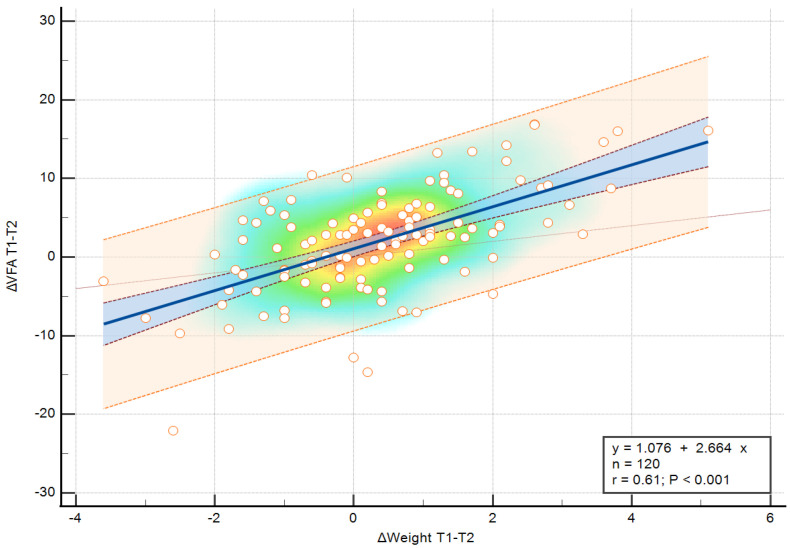
Relationship between changes in body weight (ΔWeight) and changes in visceral fat area (ΔVFA) between T1 and T2. The fitted linear relationship, including the 95% confidence interval, is shown in blue. Color shading reflects observation density and individual values are shown as points.

Linear regression analysis revealed a significant relationship between ΔWeight and ΔVFA (R^2^ = 0.370, *p* < 0.001). As seen in Figure 4, ΔWeight explained about 37% of ΔVFA variance, with an estimated increase of 2.66 cm^2^ in VFA per kilogram of weight gain.

**Figure 5:**
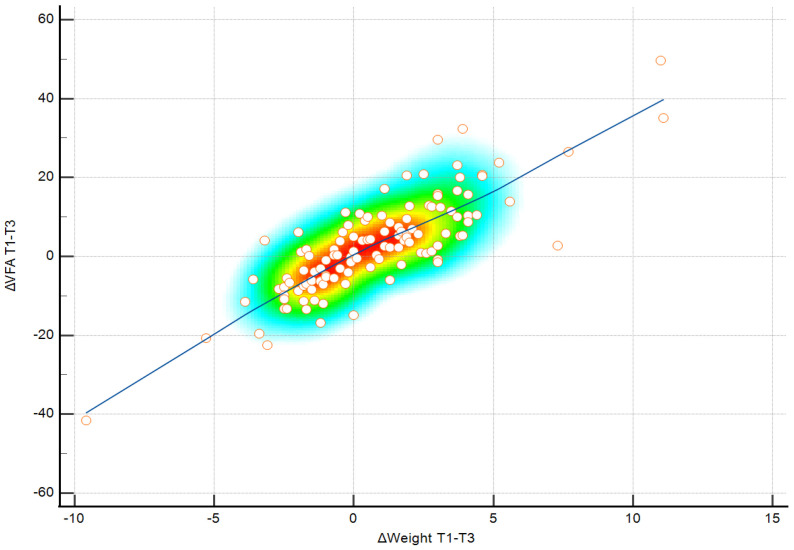
Association between changes in visceral fat area (ΔVFA) and changes in body weight (ΔWeight) over the entire one-year follow-up period (T1–T3), assessed using Spearman correlation. Color shading reflects observation density, individual values are shown as points, and the blue line represents the linear association.

**Figure 6:**
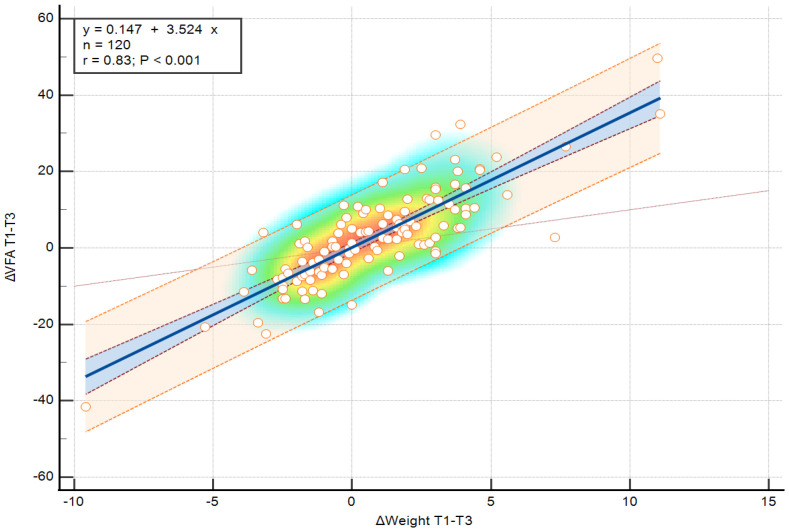
Simple linear regression illustrating the relationship between changes in visceral fat area (ΔVFA) and changes in body weight (ΔWeight) over the entire one-year follow-up period (T1–T3). The fitted linear relationship, including the 95% confidence interval, is shown in blue. Color shading reflects observation density and individual values are shown as points.

The weight gain observed during the winter holiday period represented approximately 95% of the net annual weight gain (T1–T3) at the group level. Similarly, the increase in VFA between T1 and T2 accounted for approximately 70% of the total mean VFA difference over the full follow-up interval. To further evaluate if the association between ΔWeight and ΔVFA observed during the holiday period persisted over the longer term, correlation and regression analyses were also performed for changes between T1 and T3 (Figure 5 and Figure 6). A strong positive association was identified between ΔWeight (T1–T3) and ΔVFA (T1–T3), as demonstrated by Spearman correlation analysis (ρ = 0.804, *p* < 0.001). Linear regression analysis revealed a significant relationship between ΔWeight (T1–T3) and ΔVFA (T1–T3) over the one-year follow-up period (R^2^ = 0.696, *p* < 0.001).

## 4. Discussion

The persistence of winter holiday–related changes in body composition was examined, with a particular focus on body weight and VFA assessed by BIA.

The prevalence of obesity continues to rise worldwide, but the scientific literature is still catching up in identifying the complex factors that contribute to weight gain. In this context, short-term and seasonal changes in body weight have gained attention as a possible contributor to long-term weight gain. The present discussion places these findings in the context of existing literature and explores their potential implications.

One period that has received particular attention for its contribution to annual weight gain is the end of the year. During this time, multiple cultural and social holidays are celebrated worldwide. This period is typically characterized by increased caloric intake, driven by higher food consumption, greater food availability, larger portion sizes, longer mealtimes, and more frequent social gatherings that involve shared meals [30,31,32,33]. Experimental and observational studies have consistently shown that portion size and eating context influence energy intake, with individuals consuming more food in larger portions and in social settings, a phenomenon described as social facilitation of eating [34,35,36,37]. Meal duration and environmental distractions further contribute to increased intake during festive occasions [38,39,40]. These behavioral and environmental factors are common during holidays. Several studies and reviews have examined the impact of this period on body weight and consistently report modest increases during the holiday interval. In adults, the reported weight gain typically ranges between 0.4 and 0.9 kg, and in some cohorts it persists beyond the holiday season [15,16,41].

These results are consistently reported across different countries. Prospective studies conducted in the United States have shown modest but significant increases during the Thanksgiving-Christmas-New Year period, often accounting for a substantial proportion of the annual weight gain [42,43,44,45,46]. Similar patterns have been observed internationally, including in Germany, Japan, and other European populations, despite differences in the specific holidays and their duration [30,47,48,49]. Weight gain during holiday periods appears to be a common finding, even though the number and type of holidays vary between countries. However, most available evidence comes from studies conducted in the United States and a limited number of other Western countries. As a result, it remains unclear to what extent these findings apply to populations with different cultural backgrounds and holiday patterns. Also, data on the long-term persistence of these changes remain scarce.

In Romania, the end-of-year period is marked by multiple public holidays and celebrations that extend into early January. This interval includes a sequence of national and religious holidays. Because these holidays are closely spaced and include several legal days off work, many people take time off during this interval. Consequently, the end-of-year becomes a prolonged and relatively continuous festive period across the country.

In this context, the present study provides longitudinal data on changes in body weight and body composition during the winter holiday period and their persistence over a one-year follow-up in a Romanian adult population. In line with previous studies, the winter holiday period in our cohort was associated with a modest but statistically significant increase in body weight. This change was accompanied by increases in BFM and VFA. Significant changes were also observed in WC, HC, WHR, and WHtR. Lean mass parameters and body water compartments remained largely unchanged during this period. These findings show that the winter holiday period in Romania was accompanied by measurable alterations in body composition. Beyond body weight, an important aspect is the small increase observed in VFA. While statistically significant, such a small difference must be interpreted in light of the known variability of BIA measurements [25,26,27,28,50]. Given the well-known link between visceral adiposity and cardiometabolic risk, an important question is whether these changes are transient or persist over time [20,51,52,53,54,55]. If maintained, such changes may have relevant long-term health implications, although their individual clinical significance remains uncertain. To address this question, alterations in body composition were evaluated over the one-year follow-up. After the increase observed during the winter holiday period, body weight was almost the same after a year, with no significant difference between T2 and T3. Similar patterns were observed for BFM, PBF, and VFA, with no statistically significant changes at follow-up. The increase observed during the winter holiday period was of similar magnitude with the total annual change in body weight and VFA (T1–T3). Given the interval between follow-up assessments, intermediate fluctuations cannot be excluded. Weight alterations may have occurred between T2 and T3 and were not captured by the study design, but the net annual change observed at follow-up was comparable with the changes observed after the holiday period. This pattern suggests that this short seasonal interval may have an impact on body weight and body composition similar in magnitude to that observed across the entire year.

When participants were grouped according to post-holiday weight change, no statistically significant differences were observed in baseline characteristics. Participants who maintained their body weight had a lower median baseline weight than those who lost weight or gained further weight. This difference was not statistically significant. These findings suggest that baseline body weight alone does not predict post-holiday weight trajectories, although a lower initial weight may be linked to weight maintenance after the holiday period. Also, despite clear baseline differences between sexes, both the holiday-related changes and their persistence over the one-year follow-up showed no statistically significant sex-specific differences in magnitude. Although males and females started from different absolute values, the pattern of change over time was similar. This indicates that the observed effects were not primarily driven by sex. However, given the smaller number of male participants, small sex-specific differences cannot be completely excluded. Our cohort covered a relatively broad adult age range, but exploratory analyses did not demonstrate significant associations between age and the magnitude of changes in body weight or VFA. So, in this sample, age did not appear to meaningfully influence either the holiday-related changes or their persistence over one year.

Changes in body weight were strongly associated with changes in VFA. This relationship was evident both during the winter holiday period and across the one-year follow-up. Even modest increases in body weight were accompanied by detectable increases in VFA. In line with earlier observations, these findings suggest that even small weight changes are accompanied by parallel changes in VFA, although part of this association may be explained by the way VFA is estimated by BIA, which takes body weight into account [6,21,51]. The overall time effect for body weight was not statistically significant, but small increases were observed in BFM and VFA. Body weight reflects the sum of BFM and FFM. Therefore, minor increases in BFM may occur without producing a significant change in total body weight. The significant time effects for BFM and VFA likely reflect small changes in body composition rather than substantial weight gain.

The magnitude of weight gain observed in our cohort is comparable with values reported in previous studies conducted in the United States and other European populations [16,30,46,56,57,58]. Only a limited number of studies have examined persistence beyond the immediate holiday period. Some reported that weight gain may still be present at short-term follow-up, while others showed that part of the gained weight is subsequently lost [30,56]. Longer follow-up data remain scarce. Data beyond body weight are even more limited. Some data suggest that BFM may increase despite minimal changes in total body weight, indicating that body composition can change even when weight appears stable [23]. Considering that most available evidence originates from the United States and a limited number of Western countries, we think our study provides complementary data from a Romanian adult population, where cultural patterns and holiday structure differ, and extends previous observations by examining both body weight and body composition across a longer interval.

Our findings may have relevant clinical implications. The winter holiday period represents a short but important window during which small weight gains were accompanied by parallel increases in visceral fat. The changes in body weight and VFA observed after the holiday period were similar in magnitude at the one-year follow-up assessment. Even if these changes are modest and not clinically dramatic at first glance, they deserve attention. One important observation from this study is that, in our cohort, the net change observed between T1 and T2 accounted for a large proportion of the overall T1–T3 difference in both body weight and VFA at the group level. However, given the interval between assessments, changes occurring between T2 and T3 cannot be characterized in detail. Considering that visceral adiposity is closely linked to cardiometabolic risk, preventing even modest winter holiday-related weight gain may be clinically important. Simple preventive strategies aimed at weight maintenance during this period could have a meaningful impact on long-term metabolic health, although the clinical impact of the modest changes observed in this study remains uncertain. Importantly, changes in body weight alone may underestimate these changes, highlighting the value of assessing body composition when possible.

This study has several limitations that should be acknowledged when interpreting the results. First, the study sample may not fully reflect the general adult population due to selection bias and differences in gender distribution. Participants were recruited voluntarily through social media announcements, resulting in a convenience sample rather than a population-based cohort. This approach introduces the possibility of self-selection bias, as individuals who chose to participate may have been more interested in body composition and lifestyle behaviors than the general population. The higher proportion of females further limits the generalizability of the findings. Second, the study was conducted in a specific geographic region of Romania, with participants mainly from western Romania. Winter holiday traditions, dietary habits, and social practices vary not only between countries but also across regions within the same country. Therefore, the observed effects may not fully apply to other populations with different cultural and holiday patterns. Third, participant motivation to attend the assessments may have influenced the results. All participants left the clinic with printed InBody 770 body composition reports after each measurement. Awareness of these results may have led some individuals to modify their behavior or lifestyle, which may not reflect typical patterns in the general population. Fourth, the study included only three assessment points with a gap of almost 11 months. Changes in body composition may have occurred between measurements or during other periods of the year, such as the summer holidays, which were not captured. Also, the final assessment was conducted in late November, shortly before the next winter holiday period. This timing was chosen to approximate a one year interval from baseline, but body composition changes related to upcoming festivities may begin gradually and cannot be excluded. In addition, important lifestyle factors such as physical activity, sleep, and alcohol consumption were not assessed and may have acted as confounding variables. Therefore, the present study cannot determine which specific behavioral factors contributed to the observed changes in weight and body composition. Also, the study did not collect longitudinal data on socioeconomic status, employment characteristics, or detailed environmental context. Variables related specifically to the holiday period, including residential environment during the celebrations (rural/urban) and self-reported dietary patterns over the holiday interval, were collected and analyzed in the initial T1–T2 phase of the study [24]. As these variables were intended to characterize short-term holiday-related behaviors and were comprehensively reported in the previously published analysis, they were not reintroduced in the present manuscript, which focuses on the longitudinal persistence of body composition changes across one year. Assessment of body composition was based on BIA. Although this method shows good agreement with DXA in healthy adults and is practical for repeated measurements, it is not the gold standard for body composition assessment and DXA remains the reference method [59,60,61,62]. In addition, BIA is less accurate than DXA, MRI, or CT for assessing visceral fat [61,63]. Considering the aim of the study, the use of BIA was appropriate, as imaging methods such as MRI, DXA, or CT would not be feasible or ethically appropriate. Future studies should include larger and more diverse populations, as well as more frequent assessments, to better describe weight and body composition changes across the year.

## 5. Conclusions

The study suggests that the measurable changes in body weight and body composition, including increases in visceral fat, initially observed during the winter holiday period were still present at the one-year follow-up. Although these changes are modest in absolute terms, they are similar in magnitude to the net annual difference observed at the group level. This indicates that short, recurrent periods such as the winter holidays may contribute to overall yearly changes in body weight and fat distribution. Preventive strategies focused on weight maintenance during this period could be considered, although the clinical impact of the modest changes observed remains uncertain. Moreover, assessment of body composition, rather than body weight alone, may provide a more accurate understanding of these changes. Future research with more frequent assessments throughout the year, especially before and after other holiday periods, would help clarify these trajectories and should further explore the role of dietary and physical activity patterns.

## Figures and Tables

**Table 1:** Baseline characteristics of the study participants (n = 120).

Variable	Value
Age (years)	30 (26–43.5)
Height (cm)	167.95 ± 9.01
Weight (kg)	68.25 (59.85–80.3)
BMI (kg/m^2^)	24.3 (21.6–27.3)
BFM (kg)	21.09 ± 8.27
PBF (%)	29.83 ± 8.47
FFM (kg)	46.1 (40.95–54.7)
SMM (kg)	25.3 (22.2–30.9)
TBW (l)	33.75 (30–40.05)
ECW (l)	12.8 (11.40–15.05)
ICW (l)	20.95 (18.6–25.2)
VFA (cm^2^)	98.49 ± 45.15
VFL (level)	8.5 (6–12)
WC (cm)	83.36 ± 12.31
HC (cm)	101 (95–106)
WHR	0.823 ± 0.081
WHtR	0.496 ± 0.069

Abbreviations used in the table: BMI—body mass index; BFM—body fat mass; PBF—percentage of body fat; FFM—fat-free mass; SMM—skeletal muscle mass; TBW—total body water; ICW—intracellular water; ECW—extracellular water; VFA—visceral fat area; VFL—visceral fat level; WC—waist circumference; HC—hip circumference; WHR—waist-to-hip ratio; WHtR—waist-to-height ratio.

**Table 2:** Baseline demographic and body composition characteristics by sex.

Variable	Females (n = 86)	Males (n = 34)	*p*-Value
Height (cm) ^a^	163.76 ± 6.31	178.55 ± 5.4	<0.001
Age (years) ^b^	30.0 (27.0–46.0)	29.0 (26.0–33.0)	0.246
Weight (kg) ^b^	63.05 (55.8–72.2)	84.35 (72.0–89.8)	<0.001
BMI (kg/m^2^) ^b^	23.8 (21.3–27.1)	25.45 (23.1–28.2)	0.017
BFM (kg) ^a^	22.04 ± 8.35	18.68 ± 7.66	0.044
PBF (%) ^a^	32.81 ± 7.087	22.297 ± 6.931	<0.001
FFM (kg) ^a^	43.176 ± 5.147	63.306 ± 7.803	<0.001
SMM (kg) ^a^	23.484 ± 3.023	35.905 ± 4.674	<0.001
TBW (l) ^a^	31.647 ± 3.779	46.403 ± 5.729	<0.001
ECW (l) ^a^	12.10 ± 1.479	17.33 ± 2.169	<0.001
ICW (l) ^a^	19.538 ± 2.319	29.071 ± 3.593	<0.001
VFA (cm^2^) ^a^	104.6 ± 46.44	83.03 ± 38.1	0.017
VFL (level) ^b^	9.95 (6.0–13.0)	7.76 (5.0–10.0)	0.016
WC (cm) ^a^	80.035 ± 11.375	91.77 ± 10.569	<0.001
HC (cm) ^b^	99.5 (94–105)	103.45 (100–107)	0.024
WHR ^a^	0.799 ± 0.074	0.885 ± 0.063	<0.001
WHtR ^b^	0.479 (0.442–0.532)	0.521 (0.472–0.557)	0.045

^a^ independent-sample T-test; ^b^ Mann–Whitney; Abbreviations used in the table: BMI—body mass index; BFM—body fat mass; PBF—percentage of body fat; FFM—fat-free mass; SMM—skeletal muscle mass; TBW—total body water; ICW—intracellular water; ECW—extracellular water; VFA—visceral fat area; VFL—visceral fat level; WC—waist circumference; HC—hip circumference; WHR—waist-to-hip ratio; WHtR—waist-to-height ratio.

**Table 3:** Anthropometric parameters and body composition changes between T1 and T2.

Variable	Before (T1)	After (T2)	Difference (95% CI)	*p*-Value
Weight (kg) ^a^	68.25 (59.85–80.30)	69.40 (60.45–80.35)	0.35 (0.05–0.60) ^b^	0.015
BMI (kg/m^2^) ^a^	24.3 (21.6 –27.3)	24.40 (21.7–27.25)	0.1 (0.00–0.20) ^b^	0.018
BFM (kg) ^c^	21.09 ± 8.27	21.43 ± 8.33	0.34 (0.11–0.55)	0.002
PBF (%) ^c^	29.83 ± 8.47	30.16 ± 8.53	0.32 (0.08–0.56)	0.009
FFM (kg) ^a^	46.1 (40.95–54.7)	45.9 (40.95–55.35)	0.05 (−0.20–0.25) ^b^	0.766
SMM (kg) ^a^	25.30 (22.2–30.9)	25.10 (22.2–31.15)	0.05 (−0.10–0.15) ^b^	0.582
TBW (l) ^a^	33.75 (30–40.05)	33.65 (30.0 –40.55)	0 (−0.10–0.20) ^b^	0.706
ECW (l) ^a^	12.8 (11.4–15.05)	12.9 (11.34–15.25)	0 (−0.05–0.10) ^b^	0.768
ICW (l) ^a^	20.95 (18.6–25.2)	20.75 (18.6–25.4)	0 (−0.10–0.10) ^b^	0.543
VFA (cm^2^) ^c^	98.49 ± 45.15	100.54 ± 45.67	2.04 (0.85–3.23)	<0.001
VFL (level) ^a^	8.5 (6–12)	9 (6–12)	0 (0.00–0.50) ^b^	0.009
WC (cm) ^c^	83.36 ± 12.31	84.09 ± 12.39	0.73 (0.36–1.10)	<0.001
HC (cm) ^a^	101 (95–106)	101 (96–106)	0 (0.00–0.50) ^b^	0.006
WHR ^c^	0.823 ± 0.081	0.827 ± 0.078	0.004 (0.001–0.008)	0.02
WHtR ^c^	0.496 ± 0.069	0.5 ± 0.069	0.004 (0.002–0.006)	<0.001

^a^ Wilcoxon signed-rank test; ^b^ Hodges–Lehmann median difference (95% CI); ^c^ paired T-test; Abbreviations used in the table: BMI—body mass index; BFM—body fat mass; PBF—percentage of body fat; FFM—fat-free mass; SMM—skeletal muscle mass; TBW—total body water; ICW—intracellular water; ECW—extracellular water; VFA—visceral fat area; VFL—visceral fat level; WC—waist circumference; HC—hip circumference; WHR—waist-to-hip ratio; WHtR—waist-to-height ratio.

**Table 4:** Follow-up changes from T2 to T3 in body composition and anthropometry.

Variable	T2	T3	Difference (95% CI)	*p*-Value
Weight (kg) ^a^	69.40 (60.45–80.35)	69.45 (59.75–80.9)	0.15 (−0.10–0.85) ^b^	0.124
BMI (kg/m^2^) ^a^	24.40 (21.7–27.25)	24.35 (21.85–27.35)	0.15 (−0.05–0.30) ^b^	0.109
BFM (kg) ^c^	21.43 ± 8.33	21.64 ± 8.43	0.21 (−0.19–0.62)	0.305
PBF (%) ^c^	30.16 ± 8.53	30.28 ± 8.56	0.12 (−0.29–0.53)	0.559
FFM (kg) ^a^	46.1 (40.95–54.7)	46.05 (41.35–55.8)	0.25 (0.00–0.50) ^b^	0.063
SMM (kg) ^a^	25.1 (22.2–31.15)	25.2 (22.45–31.25)	0.15 (0.00–0.30) ^b^	0.044
TBW (l) ^a^	33.65 (30–40.55)	33.5 (30.25 –40.65)	0.15 (−0.05–0.30) ^b^	0.169
ECW (l) ^a^	12.9 (11.35–15.25)	12.8 (11.4–15.35)	0 (−0.05–0.10) ^b^	0.512
ICW (l) ^a^	20.75 (18.6–25.4)	20.9 (18.7–25.5)	0.10 (0.00–0.25) ^b^	0.058
VFA (cm^2^) ^c^	100.54 ± 45.67	101.42 ± 45.98	0.88 (−1.47–3.24)	0.460
VFL (level) ^a^	9 (6–12)	9 (6–12)	0 (0.00–0.50) ^b^	0.310
WC (cm) ^c^	84.09 ± 12.39	84.30 ± 12.36	0.20 (−0.11–0.52)	0.199
HC (cm) ^a^	101 (96–106)	101 (96–106.5)	0 (0.00–0.50) ^b^	0.06
WHR ^c^	0.827 ± 0.078	0.828 ± 0.078	0.00 (0.001–0.002)	0.583
WHtR ^c^	0.5 ± 0.069	0.502 ± 0.069	0.001 (0.00–0.003)	0.191

^a^ Wilcoxon signed-rank test; ^b^ Hodges–Lehmann median difference (95% CI); ^c^ paired T-test Abbreviations used in the table: BMI—body mass index; BFM—body fat mass; PBF—percentage of body fat; FFM—fat-free mass; SMM—skeletal muscle mass; TBW—total body water; ICW—intracellular water; ECW—extracellular water; VFA—visceral fat area; VFL—visceral fat level; WC—waist circumference; HC—hip circumference; WHR—waist-to-hip ratio; WHtR—waist-to-height ratio.

**Table 5:** Longitudinal changes in primary outcomes across the three assessment points (T1–T2–T3).

Variable	T1	T2	T3	Overall *p*-Value
Weight (kg) ^a^	68.25 (59.85–80.3)	69.40 (60.45–80.35)	69.45 (59.75–80.9)	0.122
BFM (kg) ^b^	21.09 ± 8.27	21.43 ± 8.33	21.64 ± 8.43	0.020
VFA (cm^2^) ^b^	98.49 ± 45.15	100.54 ± 45.67	101.42 ± 45.98	0.025

^a^ Friedman test; ^b^ Repeated-measures ANOVA (Greenhouse–Geisser correction); Abbreviations used in the table: BFM—body fat mass; VFA—visceral fat area.

**Table 6:** Baseline characteristics according to post-holiday weight-change categories during follow-up (T2–T3).

Variable	Weight Loss (<−1 kg)	Weight Maintenance (±1 kg)	Further Weight gain (>+1 kg)	*p*-Value
Participants, n (%)	35 (29.2%)	41 (34.2%)	44 (36.7%)	-
Age (years) ^a^	30 (25.3–43.0)	35 (27.0–49.0)	28 (26.5–39.0)	0.125
Weight at baseline (kg) ^a^	68.5 (61.3–83.7)	63.9 (59.1–84.6)	69.8 (60.7–78.3)	0.706
VFA at baseline (cm^2^) ^a^	103.21 ± 44.02	100.25 ± 52.15	93.10 ± 39.09	0.609
BFM at baseline (kg) ^a^	21.78 ± 7.728	21.30 ± 9.747	20.34 ± 7.265	0.681
Females, n (%) ^b^	24 (68.6%)	32 (78.0%)	30 (68.2%)	0.535
Males, n (%) ^b^	11 (31.4%)	9 (22.0%)	14 (31.8%)	0.535

^a^ Kruskal–Wallis test; ^b^ Chi-squared test. Abbreviations used in the table: VFA—visceral fat area; BFM—body fat mass.

## Data Availability

The data of this study can be obtained from the corresponding author upon reasonable request.

## Supplementary Materials

The following supporting information can be downloaded at: https://www.mdpi.com/article/10.3390/medicina62030511/s1, Table S1. Baseline characteristics of completers vs dropouts; Table S2. Sex differences in holiday-related changes (T1–T2); Table S3. Sex differences in follow-up changes (T2–T3); Table S4. Longitudinal changes in body composition and anthropometric parameters across the three assessment points (T1–T2–T3).
