# Supplementary material for: Persistence of Body Composition Changes Observed During the Winter Holiday Period: A Three-Time-Point, One-Year Longitudinal Study
Source: Medicina (Kaunas). 2026 Mar 10;62(3):511. doi: 10.3390/medicina62030511 (PMC13028301; doi:10.3390/medicina62030511)
Supplement: Supplementary file 1 [file medicina-62-00511-s001.zip › medicina-4165112-supplementary.pdf]

medicina

MDPI

Supplementary Table S1. Baseline characteristics of completers vs dropouts.

Variable	Completers (n = 120)	Dropouts (n = 48)	*p*-value
Age (years)[a]	30 (26–43.5)	29.0 (27.0 - 45.0)	0.709
Female n (%)[b]	86 (71,7%)	40 (83,3%)	0.447
Height (cm)[c]	167.95 ± 9.01	168.16 ± 8.4	0.887
Weight (kg)[a]	68.25 (59.85–80.3)	70.15 (61.0 - 85.15)	0.234
BMI (kg/m^2)[a]	24.3 (21.6–27.3)	25.2 (21.6-29.35)	0.193
BFM (kg)[c]	21.09 ± 8.27	23.12 ± 11.20	0.083
PBF (%)[c]	29.83 ± 8.47	31.12 ± 9.27	0.098
VFA (cm^2)[c]	98.49 ± 45.15	99.1 ± 46.28	0.512
WC (cm)[c]	83.36 ± 12.31	84.15 ± 11.78	0.412
HC (cm)[a]	101 (95–106)	104 (95.5- 111.5)	0.080

[a] Mann–Whitney; [b] Chi-squared test; [c] independent-sample T-test; Abbreviations used in the table: BMI – body mass index; BFM – body fat mass; PBF – percentage of body fat; VFA – visceral fat area; WC – waist circumference; HC – hip circumference.

Supplementary Table S2. Sex differences in holiday-related changes (T1–T2).

Variable	Δ Females (T2–T1)	Δ Males (T2–T1)	P-value
Weight (kg)[a]	0.20 (-0.8 – 0.9)	0.75 (-0.40 – 2.1)	0.056
BMI (kg/m^2)[a]	0.1 (–0.3 – 0.4)	0.25 (–0.2 – 0.7)	0.094
BFM (kg)[b]	0.232 ± 1.154	0.605 ± 1.350	0.161
PBF (%)[b]	0.264 ± 1.344	0.461 ± 1.309	0.462
FFM (kg)[a]	–0.2 (–0.6 – 0.5)	0.55 (–0.6 – 1.2)	0.090
SMM (kg)[a]	–0.1 (–0.4 – 0.3)	0.35 (–0.3 – 0.8)	0.055
TBW (l)[a]	–0.1 (–0.4 – 0.4)	0.3 (–0.5 – 0.9)	0.090
ECW (l)[a]	0 (–0.2 – 0.2)	0.1 (–0.2 – 0.4)	0.279
ICW (l)[a]	–0.1 (–0.3 – 0.3)	0.3 (–0.3 – 0.5)	0.067
VFA (cm^2)[b]	1.76 ± 6.68	2.77 ± 6.35	0.443
VFL (level)[a]	0 (0 – 1)	0 (0 – 1)	0.161
WC (cm)[b]	0.657 ± 2.231	0.926 ± 1.538	0.452
HC (cm)[a]	0 (0 – 1)	1 (0 – 2)	0.167
WHR[b]	0.004 ± 0.023	0.005 ± 0.011	0.678
WHtR[b]	0.004 ± 0.013	0.005 ± 0.008	0.568

[a] Mann–Whitney; [b] independent-sample T-test; Abbreviations used in the table: BMI – body mass index; BFM – body fat mass; PBF – percentage of body fat; FFM – fat-free mass; SMM – skeletal muscle mass; TBW – total body water; ICW – intracellular water; ECW – extracellular water; VFA – visceral fat area; VFL – visceral fat level; WC – waist circumference; HC – hip circumference; WHR – waist-to-hip ratio; WHtR – waist-to-height ratio.

 https://doi.org/10.3390/medicina62030511

Supplementary Table S3. Sex differences in follow-up changes (T2- T3).

Variable	Δ Females (T2–T1)	Δ Males (T2–T1)	P-value
Weight (kg)[a]	0.10 (-1.2 – 1.6)	0.65 (-1.3 – 3.0)	0.493
BMI (kg/m^2)[a]	0 (–0.5 – 0.6)	0.2 (–0.4 – 0.9)	0.504
BFM (kg)[b]	0.012 ± 2.282	0.270 ± 2.875	0.642
PBF (%)[b]	-0.118 ± 2.74	0.102 ± 2.42	0.665
FFM (kg)[a]	0.25 (–0.7 – 1)	0.80 (–0.4 – 1.2)	0.226
SMM (kg)[a]	0.15 (–0.5 – 0.7)	0.5 (–0.3 – 0.8)	0.224
TBW (l)[a]	0.1 (–0.6– 0.7)	0.55 (–0.5 – 0.9)	0.248
ECW (l)[a]	0 (–0.2 – 0.3)	0.1 (–0.2 – 0.4)	0.333
ICW (l)[a]	0.1 (–0.4 – 0.5)	0.4 (–0.3 – 0.6)	0.198
VFA (cm^2)[b]	0.56 ± 14.75	1.102 ± 14.78	0.579
VFL (level)[a]	0 (-1 – 1)	0 (-1 – 1)	0.740
WC (cm)[b]	0.290 ± 1.585	0.441 ± 1.987	0.694
HC (cm)[a]	0 (0 – 1)	1 (0 – 1)	0.394
WHR[b]	-0.007 ± 0.073	0 ± 0.011	0.390
WHtR[b]	0.001 ± 0.009	0.002 ± 0.011	0.753

[a] Mann–Whitney; [b] independent-sample T-test; Abbreviations used in the table: BMI – body mass index; BFM – body fat mass; PBF – percentage of body fat; FFM – fat-free mass; SMM – skeletal muscle mass; TBW – total body water; ICW – intracellular water; ECW – extracellular water; VFA – visceral fat area; VFL – visceral fat level; WC – waist circumference; HC – hip circumference; WHR – waist-to-hip ratio; WHtR – waist-to-height ratio.

Supplementary Table S4. Longitudinal changes in body composition and anthropometric parameters across the three assessment points (T1–T2–T3).

Variable	T1	T2	T3	Overall *p*-value
BMI (kg/m^2)[a]	24.3 (21.6–27.3)	24.40 (21.7 – 27.25)	24.35 (21.85 – 27.35)	0.096
PBF (%)[b]	29.83 ± 8.47	30.16 ± 8.53	30.28 ± 8.56	0.058
FFM (kg)[a]	46.1 (40.95–54.7)	46.1 (40.95–54.7)	46.05 (41.35 – 55.8)	0.146
SMM (kg)[a]	25.3 (22.2–30.9)	25.1 (22.2 – 31.15)	25.2 (22.45 – 31.25)	0.040
TBW (l)[a]	33.75 (30–40.05)	33.65 (30–40.55)	33.5 (30.25 – 40.65)	0.358
ECW (l)[a]	12.8 (11.40–15.05)	12.9 (11.35–15.25)	12.8 (11.4 – 15.35)	0.914
ICW (l)[a]	20.95 (18.6–25.2)	20.75 (18.6–25.4)	20.9 (18.7 – 25.5)	0.037
VFL (level)[a]	8.5 (6–12)	9 (6 – 12)	9 (6 – 12)	0.016
WC (cm)[b]	83.36 ± 12.31	84.09 ± 12.39	84.30 ± 12.36	< 0.001
HC (cm)[a]	101 (95–106)	101 (96 – 106)	101 (96 – 106.5)	0.001
WHR[b]	0.823 ± 0.081	0.827 ± 0.078	0.828 ± 0.078	0.018
WHtR[b]	0.496 ± 0.069	0.5 ± 0.069	0.502 ± 0.069	< 0.001

[a] Friedman test; [b] Repeated-measures ANOVA (Greenhouse–Geisser correction); Abbreviations used in the table: BMI – body mass index; BFM – body fat mass; PBF – percentage of body fat; FFM – fat-free mass; SMM – skeletal muscle mass; TBW – total body water; ICW – intracellular water; ECW – extracellular water; VFA – visceral fat area; VFL – visceral fat level; WC – waist circumference; HC – hip circumference; WHR – waist-to-hip ratio; WHtR – waist-to-height ratio.
